# Patellar Dislocation and Fracture After Medial Patellofemoral Ligament Reconstruction in a Patient With Osteogenesis Imperfecta

**DOI:** 10.31486/toj.21.0025

**Published:** 2022

**Authors:** Colin J. Carroll, Michael Nammour, Jeffrey Reese, Lacey Lavie, Michael Warren, Sean Waldron

**Affiliations:** ^1^ The University of Queensland Faculty of Medicine, Ochsner Clinical School, New Orleans, LA; ^2^ Department of Orthopedic Surgery, Ochsner Clinic Foundation, New Orleans, LA

**Keywords:** *Osteogenesis imperfecta*, *patella*, *patellar dislocation*, *patellar ligament*

## Abstract

**Background:** Patellar instability is a common orthopedic condition in the pediatric population. Many factors contribute to patellar instability, including trochlear dysplasia. However, patellar instability and its treatments are not well documented in the literature for patients with osteogenesis imperfecta.

**Case Report:** After medial patellofemoral ligament (MPFL) reconstruction, a 17-year-old male with osteogenesis imperfecta had a patellar dislocation that resulted in a patellar fracture. The patient subsequently had a revision of his MPFL reconstruction, and at 2½ years postoperation has had no episodes of recurrent patellar instability.

**Conclusion:** The combination of bone fragility, trochlear dysplasia, and strength of the allograft used for MPFL reconstruction compared to the patient's bone strength led to dislocation and patellar fracture. Research into alternative methods for patellar fixation and postoperative physical therapy protocols for patients with osteogenesis imperfecta is needed. Special considerations must be made for this patient population.

## INTRODUCTION

Patellar instability is a common orthopedic condition in the pediatric population.^1-6^ Many factors contribute to patellar instability, including trochlear dysplasia.^1-5^ Trochlear dysplasia refers to an abnormality of the trochlear groove of the femur in which the trochlear groove is more shallow compared to normal anatomy.^1-5^ No studies address patellar instability, medial patellofemoral ligament (MPFL) reconstruction, or postoperative complications among patients with osteogenesis imperfecta. Osteogenesis imperfecta is an inherited disorder that results in a defect in the production of type 1 collagen, leading to weak and malformed bones.^7-11^

We report the case of a patient with osteogenesis imperfecta and a history of patellar instability who underwent MPFL reconstruction and experienced a patellar fracture postoperatively.

## CASE REPORT

**Figure 1.**
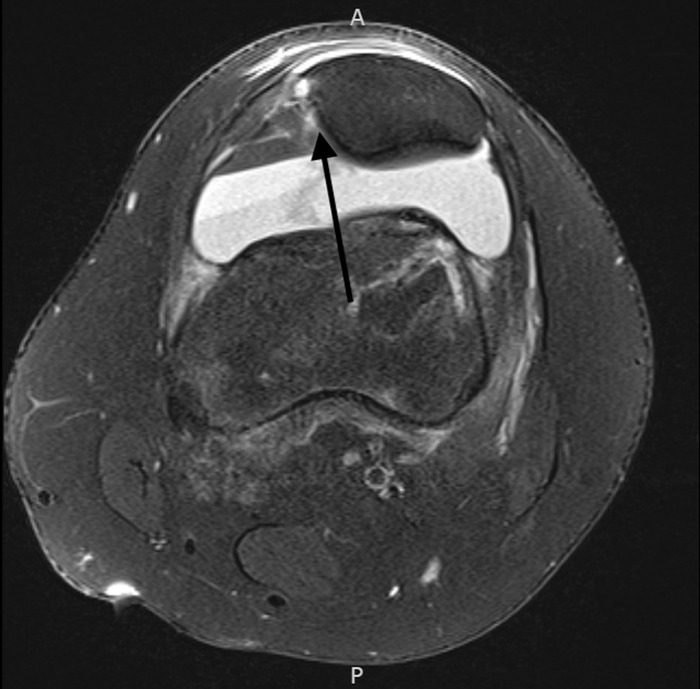
Axial fat-suppressed proton density-weighted magnetic resonance imaging from the initial evaluation in the emergency department shows disruption to the medial patellofemoral ligament-vastus medialis oblique complex in the left knee (arrow).

A 17-year-old male with an unknown subtype of osteogenesis imperfecta and a history of left patellar dislocation presented to the emergency department (ED) with left knee pain and left forearm pain after a fall from standing height. He noticed that during the fall his patella dislocated laterally and spontaneously reduced. This incident was his second patellar dislocation. His first patellar dislocation resulted in an MPFL tear that was treated nonoperatively. Upon evaluation in the ED, radiographs of the left knee showed no fracture or dislocation. Further workup with magnetic resonance imaging (MRI) demonstrated a nondisplaced osteochondral injury to the medial patella, as well as a torn medial retinaculum involving the MPFL and vastus medialis oblique (VMO) (Figure 1). In addition, a displaced left ulnar shaft fracture was found on upper extremity radiographs. The decision was made to proceed with open reduction and internal fixation of the left ulnar shaft fracture and left knee MPFL reconstruction in 1 week.

Arthroscopy at the time of surgery revealed a large medial patellar defect and a 2 × 2-cm loose body. Two cartilage biopsies were taken and saved for possible future autologous cartilage grafting. The patellar instability was treated with reconstruction of the MPFL with a gracilis tendon allograft. At the superior medial border of the patella, a 1-cm transverse drill hole was made from medial to lateral, and a second drill hole was made from anterior to posterior, connecting to the first drill hole with a 1-cm bone bridge. The MPFL insertion point on the femur was identified using fluoroscopy. A soft tissue tunnel was made between the VMO and knee capsule, and the graft was passed through the patellar tunnel. The 2 free ends were sutured together using #2 SutureLoop (CONMED Corp) and passed through the soft tissue tunnel and out the medial incision. The graft was then placed in the femoral tunnel. The graft was tensioned at 30° of flexion, and a 6-mm GENESYS Matryx biocomposite screw (CONMED Corp) was placed in the distal femoral tunnel in interference-fit fashion. Patellar tracking was assessed, and the patella was noted to track appropriately. The left ulnar shaft fracture was reduced, and a 6-hole locking plate was used for fixation. The left knee was placed in a hinged knee brace locked in extension.

The patient had moderate to severe pain in his upper and lower extremities on postoperative day (POD) 1 that required oral and intravenous opioids and acetaminophen for pain control. He was discharged home on POD 2 after clearance by the physical therapy team and with adequate pain control medication: naproxen 500 mg twice daily and oxycodone-acetaminophen 10/325 mg as needed every 4 hours. At his 3-week follow-up, the patient appeared to be doing well, with physical examination demonstrating a stable patella with knee range of motion. He was referred to physical therapy. Six weeks postoperatively, the patient reported no issues with the stability of his patella.

**Figure 2.**
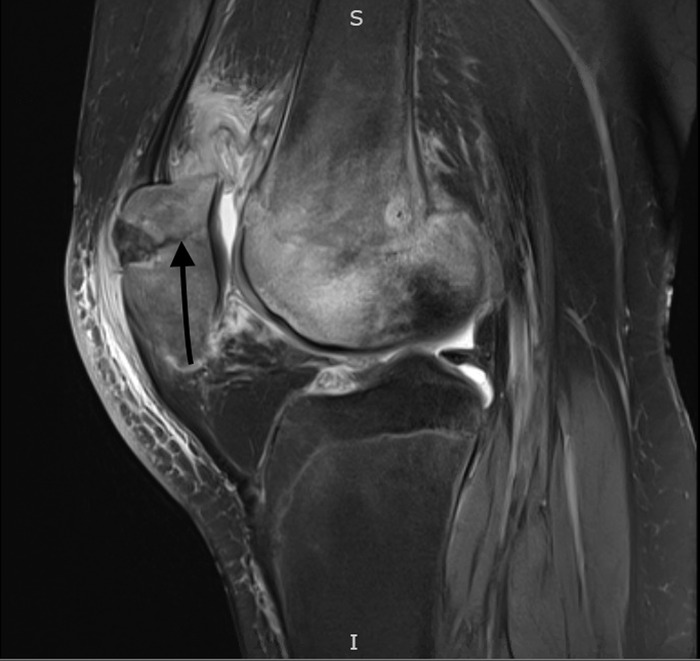
Sagittal fat-suppressed proton density-weighted magnetic resonance imaging shows a left transverse patellar fracture (arrow) after the dislocation occurred at physical therapy.

Two days following his 6-week follow-up, the patient was performing a single leg squat exercise to 45°, as instructed by the physical therapist, when his operative patella dislocated and reduced spontaneously. Radiographs in the ED revealed no new findings. MRI revealed a transverse patellar fracture along the MPFL bone anchor (Figure 2). The patient was taken back to the operating room, and arthroscopic evaluation demonstrated the femoral insertion site to be well fixated; however, a fracture compromising the patellar tunnel was noted, and the intact graft was removed from the patellar insertion site. A #2 SutureLoop was passed through the looped end of the graft, and 2 Beath pins were drilled from medial to lateral across the patella with a bone bridge between. The Beath pins were used to pass the 2 ends of the suture through the patella. An incision was made over the lateral patella, and the knee was held at 30° while the suture was tied on the lateral aspect of the patella, leading to appropriate anatomic alignment. As backup fixation, a Y-Knot (2.8 mm, 575N strength) all-suture anchor (CONMED Corp) was placed in the anterior patella, and the sutures were passed through the graft and tied down to keep the graft in position. Under arthroscopic evaluation, the knee was taken through range of motion, and patellar tracking was noted to be appropriate. The patient was placed in a hinged knee brace locked in extension and was discharged home the same day of surgery. He was given a similar home medication protocol for pain control as for his previous surgery and was asked to follow up in 1 week. The patient was doing appropriately well at his 1-, 3- and 8-week follow-ups, with normal patellar tracking and minimal pain with range of motion. The patient elected not to go to formal physical therapy postoperatively.

**Figure 3.**
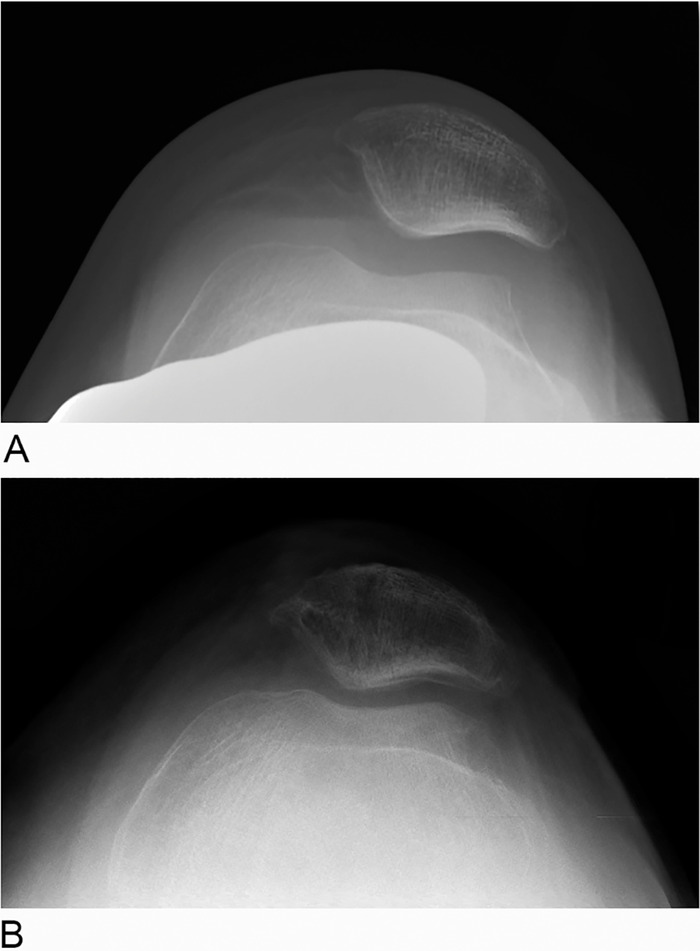
(A) Preoperative merchant view x-ray of the patient's left knee demonstrates maltracking. (B) Postoperative merchant view x-ray of the patient's left knee demonstrates improvement in articular constraint and tracking.

At 2½ years postoperatively, the patient has not had any complications since the revision MPFL reconstruction. Radiographs from before the patient's first operation (before the dislocation during physical therapy) and after the revision show the improvement in patellar tracking (Figure 3).

## DISCUSSION

**Figure 4.**
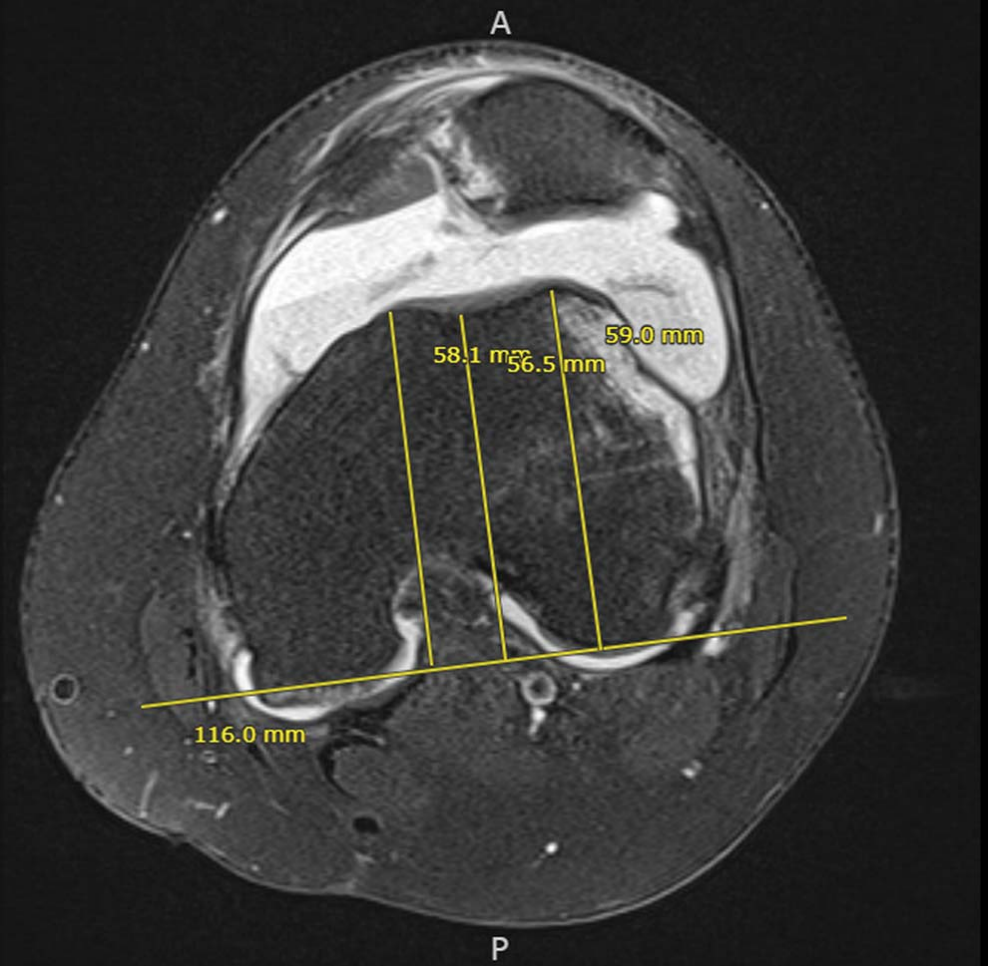
Axial fat-suppressed proton density-weighted magnetic resonance imaging (MRI) shows trochlear dysplasia and the measurements taken for determining the trochlear depth ([59 + 58.1] ÷ 2) – 56.5 = 2.05 mm. An axial MRI 3 cm above the joint line is needed to calculate the trochlear depth. The average distance of the medial and lateral facets from a line tangential to the femoral condyles is subtracted from the distance of the trochlear groove to that same tangential line. A value <3 mm is considered shallow and consistent with trochlear dysplasia.

**Figure 5.**
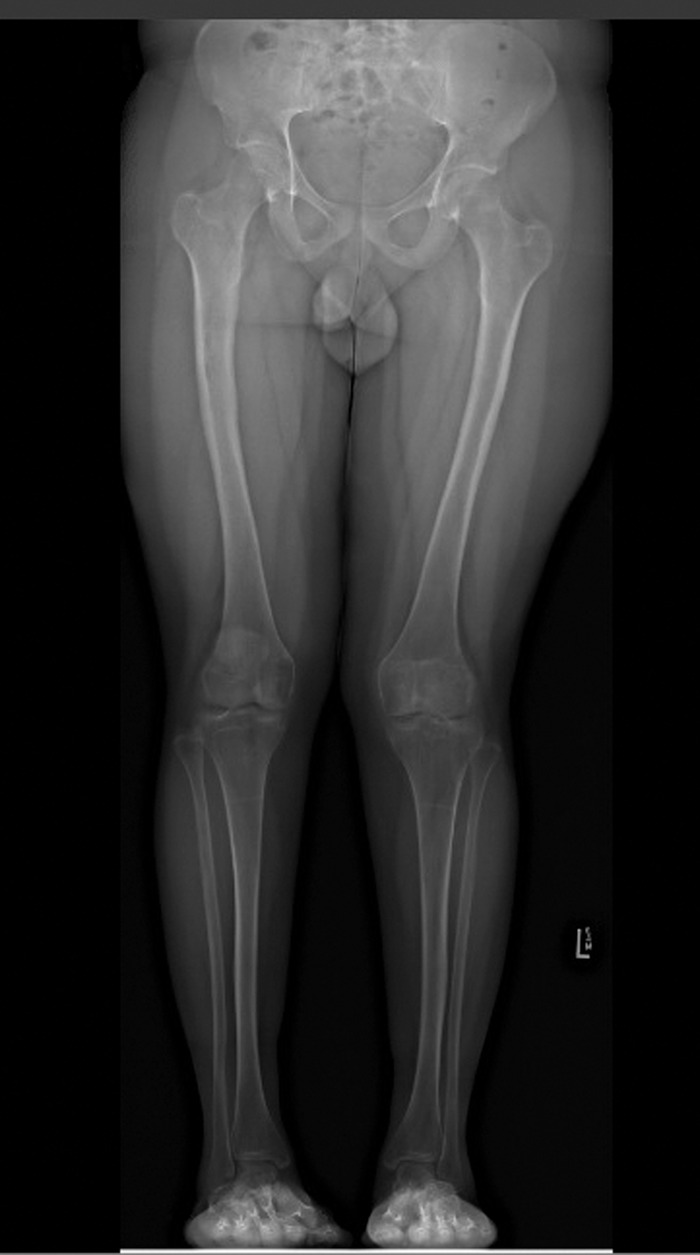
Anterior posterior hip-to-ankle x-ray demonstrates mild genu valgum.

Patellar instability, a common orthopedic condition in the pediatric population, affects up to 38% of skeletally immature patients.^1-6^ Numerous factors contribute to patellar instability, including increased Q angle, ligamentous laxity, patella alta, trochlear dysplasia, external tibial torsion, and genu valgum.^1-5^ However, one of the most significant factors affecting instability is trochlear dysplasia.^2,6^ Without the bony constraint of a well-aligned trochlea, the patella dislocates at an increased rate.^2,6^ Lewallen et al demonstrated that patients with trochlear dysplasia had a 69% risk (hazard ratio of 3.3) of redislocation with nonoperative management.^6^ Our patient had a trochlear depth of 2.05 mm, which is abnormally shallow, thus resulting in a high risk of dislocation because of the lack of articular constraint (Figure 4). Standing hip-to-ankle films showed that the patient had mild genu valgum (Figure 5). The combination of trochlear dysplasia and mild genu valgum predisposed this patient to patellar instability.

Osteogenesis imperfecta is commonly associated with skeletal abnormalities, including bone and connective tissue defects.^7-11^ Studies that specifically assessed joint hypermobility in the spine found that 30% to 70% of patients with osteogenesis imperfecta have joint hypermobility.^8,11^ While the literature documents injuries including patellar and Achilles tendon ruptures,^12,13^ to our knowledge, no research regarding patellar instability in the osteogenesis imperfecta population has been published. Our patient showed patellar instability with his history of dislocations and subluxations but did not have hypermobility in other joints on examination, making ligamentous laxity an unlikely factor.

Despite the gap in the literature regarding the influence of osteogenesis imperfecta on patellar instability, the concept of the medial patellofemoral complex has gained traction in recent (2013-2019) literature.^14-18^ The MPFL is no longer thought of as an isolated ligament with simple attachments to the femur and patella but rather as a broad, fan-shaped structure with numerous attachments.^14,17,18^ While the origin of the MPFL commonly attaches in a triangular fashion to the medial gastrocnemius tubercle, the medial femoral epicondyle, and the adductor tubercle, the insertion is more variable.^14,17,18^ Variations in the percentage of fibers attaching to the patella, as well as to the deep quadriceps tendon, have been reported.^14,19^ The integrity of the medial patellofemoral complex is vital for patellar stability, and disruption increases the risk of lateral dislocation.^14^ Disruption of this integral structure of the knee is an indication for surgical repair and reconstruction, especially in patients with recurrent patellar instability.^14,20-23^

MPFL reconstruction with gracilis allograft is a well-documented standard technique.^24-27^ Following the primary reconstruction and subsequent dislocation, the MPFL graft remained intact in our patient. However, the fracture of the patella compromised the bony fixation at the tunnel. Varying rates of recurrent instability after reconstruction have been cited, some as high as 8.1%.^25,26,28^ Patellar fractures, although rare, are a known complication following MPFL reconstruction. Schiphouwer et al reported that 3.6% of their patients sustained patellar fractures without adequate trauma after MPFL reconstruction using the technique of 2 transverse patellar tunnels.^28^ Shah et al reported that 4 of 429 patients who underwent a single or double transverse tunnel had patellar fractures postoperatively, but only 1 of the fractures was atraumatic.^20^ In our case, the patient had a dislocation while performing a partial single leg squat exercise to 45° just over 6 weeks postoperatively. Lightsey et al investigated physical therapy protocols after MPFL reconstruction and found a large range of start dates for single-leg squats: from week 4 to week 19, with a mean of week 14.^29^ A slower progression in the physical therapy protocol may need to be implemented for future patients with osteogenesis imperfecta undergoing MPFL reconstruction. Single-leg squats 6 weeks postoperatively is generally not part of the rehabilitation protocol for MPFL reconstruction, and this information should be made clear to future patients.

The method used for the initial surgery, drilling 2 tunnels at right angles to each other with a bone bridge, provided inadequate fixation because of the patient's history of osteogenesis imperfecta. This technique, however, is adequate for healthy patients. In patients with osteogenesis imperfecta, we recommend a technique relying on 2 transverse tunnels and suture anchors in each tunnel for patellar fixation. Another option for surgeons who prefer to use a bone bridge is to drill a longer transverse tunnel.

For patients with osteogenesis imperfecta who have patellar instability, other options can be considered, such as medial retinacular advancement and medial quadriceps tendon-femoral ligament repair instead of MPFL reconstruction to decrease the force across the patella. Trochleoplasty for trochlear dysplasia has been documented in the literature but has inconsistent results.^30^

## CONCLUSION

While patellar fractures are a known risk after MPFL reconstruction, the patient in this case had a higher risk of fracture secondary to his diagnosis of osteogenesis imperfecta. The combination of bone fragility and trochlear dysplasia, as well as the strength of the allograft compared to this patient's bone strength, contributed to the subsequent dislocation and patellar fracture. A slow progression through physical therapy and the use of surgical procedures other than MPFL reconstruction may decrease the risk of fractures in patients with osteogenesis imperfecta. Although the literature is inconclusive, further investigation into alternative surgical techniques and physical therapy protocols for patellar instability in both the osteogenesis imperfecta and general populations is necessary for improving future care.

## Notes

*This article meets the Accreditation Council for Graduate Medical Education and the American Board of Medical Specialties Maintenance of Certification competencies for Patient Care and Medical Knowledge.*
